# Co-Overexpression of GEP100 and AMAP1 Proteins Correlates with Rapid Local Recurrence after Breast Conservative Therapy

**DOI:** 10.1371/journal.pone.0076791

**Published:** 2013-10-07

**Authors:** Rumiko Kinoshita, Jin-Min Nam, Yoichi M. Ito, Kanako C. Hatanaka, Ari Hashimoto, Haruka Handa, Yutaro Otsuka, Shigeru Hashimoto, Yasuhito Onodera, Mitsuchika Hosoda, Shunsuke Onodera, Shinichi Shimizu, Shinya Tanaka, Hiroki Shirato, Mishie Tanino, Hisataka Sabe

**Affiliations:** 1 Department of Radiation Medicine, Hokkaido University Graduate School of Medicine, Sapporo, Hokkaido, Japan; 2 Department of Biostatistics, Hokkaido University Graduate School of Medicine, Sapporo, Hokkaido, Japan; 3 Department of Surgical Pathology, Hokkaido University Hospital, Sapporo, Hokkaido, Japan; 4 Department of Molecular Biology, Hokkaido University Graduate School of Medicine, Sapporo, Hokkaido, Japan; 5 Department of Breast and Endocrine Surgery, Hokkaido University Hospital, Sapporo, Hokkaido, Japan; 6 Laboratory of Cancer Research, Department of Pathology, Hokkaido University Graduate School of Medicine, Sapporo, Hokkaido, Japan; University of Chicago, United States of America

## Abstract

A major problem of current cancer research and therapy is prediction of tumor recurrence after initial treatment, rather than the simple biological characterization of the malignancy and proliferative properties of tumors. Breast conservation therapy (BCT) is a well-approved, standard treatment for patients with early stages of breast cancer, which consists of lumpectomy and whole-breast irradiation. In spite of extensive studies, only 'age' and 'Ki-67 positivity' have been identified to be well correlated with local recurrence after BCT. An Arf6 pathway, activated by GEP100 under receptor tyrosine kinases (RTKs) and employs AMAP1 as its effector, is crucial for invasion and metastasis of some breast cancer cells. This pathway activates β1 integrins and perturbs E-cadherin-based adhesions, hence appears to be integral for epithelial-mesenchymal transdifferentiation (EMT). We here show that expression of the Arf6 pathway components statistically correlates with rapid local recurrence after BCT. We retrospectively analyzed four hundred seventy-nine patients who received BCT in Hokkaido University Hospital, and found 20 patients had local recurrence. We then analyzed pathological samples of patients who experienced local recurrence by use of Kaplan-Meier analysis, Stepwise regression analysis and the t-test, coupled with immunostaining, and found that co-overexpression of GEP100 and AMAP1 correlates with rapidity of the local recurrence. Their margin-status, node-positivity, and estrogen receptor (ER)- or progesterone receptor (PgR)-positivity did not correlated with the rapidity. This study is the first to show that expression of a certain set of proteins correlates with the rapidity of local recurrence. Our results are useful not only for prediction, but highlight the possibility of developing novel strategies to block local recurrence. We also discuss why mRNAs encoding these proteins have not been identified to correlate with local recurrence by previous conventional gene expression profiling analyses.

## Introduction

A major problem of current cancer research and therapy is prediction of tumor recurrence after initial treatment, rather than the simple biological characterization of the malignancy and proliferative properties of tumors. Breast conservation therapy (BCT) is a well approved, standard treatment for patients with early stages of breast cancer [1-3], which consists of lumpectomy and whole-breast irradiation. Studies of relatively long years of follow-up have shown that 8.8 to 20% of breast cancer patients show local recurrence after BCT. Several factors, such as young age and high expression levels of Ki-67 antigen, a nuclear marker of cell proliferation, have been recognized to be risk factors for local recurrence after BCT [4]. Local recurrence after BCT has also been reported to vary according to 5 molecular subtypes of breast cancer, that were classified based on their gene expression signatures [5,6]. Surgical margin status, nodal status and tumor grades were also reported to be correlated with local recurrence after BCT [7,8].

Identification of gene expression signatures, as well as protein biomarkers besides Ki-67, predictive for local recurrence after BCT has been unsuccessful, while gene expression signatures indicative of malignant phenotypes of tumors and predictive for distant metastases and patient survival have been identified, though among many failures [9]. For example, the Mamma Print (Agendia, Amsterdam, the Netherlands) was found to be superior to clinico-pathological assessment in predicting distant metastases and overall survival [10-12], and has been approved by the US Food and Drug Administration. However, this 70-gene profile has turned out to be poor at predicting local recurrence, with a positive prediction value of 18% [13]. By analyzing datasets of the gene expression profiles, genes related to the wound-response signature [14] was reported to show a significant association with local recurrence after BCT [15]. This gene signature, however, was not confirmed by a following study from the same research group [13]. Moreover, recent studies in which large numbers of patients were analyzed including their gene expression profiles, age was again found to be the only independent predictor of local recurrence after BCT in multivariate analysis [13,16].

We have shown previously that the Arf6 pathway is crucial in promoting the invasion and metastasis of some breast cancer cells [17-19]. In this pathway, Arf6 is activated by GEP100 (also called BRAG2), a guanine nucleotide exchanging factor (GEF) for Arf-GTPases, and the active form of Arf6 then employs AMAP1 (DDEF1 or ASAP1) as its downstream effector. In this pathway GEP100, via its pleckstrin homology (PH) domain, directly binds to certain phosphorylated tyrosines of ligand-activated receptor tyrosine kinases (RTKs), such as EGFR (epidermal growth factor receptor) and HER2 (human epidermal growth factor receptor 2) [19]. Co-expression of GEP100 with EGFR correlates statistically with the malignant phenotypes of primary tumors of the human breast [19]. High expression levels of AMAP1 protein expression also correlate with the malignant phenotypes [18]. Our studies moreover have suggested that this Arf6 pathway may present 40-80% of invasive and malignant primary tumors of the human breast [18,19].

Malignancy development of tumor cells with an epithelial origin, in most cases, involves their transition into mesenchymal phenotypes (i.e., epithelial-mesenchymal transition: EMT). Activation of some integrins to form altered and robust interaction with their stromas, and disruption of E-cadherin-based cell-cell adhesion to allow cell detachment from their neighbors are steps necessary to proceed EMT, in order to make cells to be highly motile and invasive [20]. Activation of Arf6 has been shown to disrupt E-cadherin-based cell-cell adhesion [21]. We have shown that activation of Arf6 by GEP100, but not by other GEFs for Arf6, perturbs formation of E-cadherin-based cell-cell adhesion of breast cancer cells [19], in which AMAP1 is also essential (will be published elsewhere). AMAP1 moreover binds to protein kinase D2 (PRKD2) to make a complex with β1 integrins. Through this binding, the Arf6-AMAP1 pathway acts to promote recycling of these integrins to enhance invasiveness [22]. It has been shown that gain-of-function mutants of p53 convert some breast cancer cells into possessing mesenchymal phenotypes [23]. We have found that gain-of-function mutations of p53 are necessary to generate and activate the RTKs-GEP100-Arf6-AMAP1 pathway (will be published elsewhere). Therefore, the RTKs-GEP100-Arf6-AMAP1 pathway appears to be the pathway that executes the EMT of some breast cancer cells in response to genome alterations and RTK activation [24] (will be published elsewhere).

Protein expression of both Arf6 and AMAP1 is very high in highly-invasive breast cancer cells, but not in weakly- and non-invasive breast cancer cells and normal mammary epithelial cells [17,18]. Interestingly, overexpression of these proteins is not at all related to their mRNA levels and seems to be regulated post-transcriptionally [17,18]. Under physiological condition, Arf6 and AMAP1 proteins are both highly expressed in vesicular endothelial cells upon vesicular endothelial growth factor (VEGF) stimulation; and the GEP100-Arf6-AMAP1 pathway is crucial for VEGF-, as well as tumor-induced angiogenesis *in vivo* and *in vitro* [25].

Local recurrence of tumors after BCT may not be only a result of tumor cells left behind upon surgery, but dissemination of cancer cells before the surgery or even during their pre-cancerous stages [26] is very likely to be essential for local recurrence. Resistance of tumor cells to inonizing radiation seems to be another major factor contributing to local recurrence after BCT. Most breast tumors arise from ductal epithelial cells; hence the EMT conversion of transformed mammary ductal epithelial cells, at least transiently, is thought to be a prerequite for the transformed cells to be disseminated from the ductal structure. Moreover, β1 integrins, which are activated by the RTKs-GEP100-Arf6-AMAP1 pathway, are the major factor that render radio-resistance to breast cancer cells [27,28].

We here sought to investigate whether the presence of the RTKs-GEP100-Arf6-AMAP1 pathway is correlated with local recurrence after BCT. We also examined the expression of EGFR, HER2, ER and PgR. Node-positivity and surgical margin status of resected specimens, as well as age of patients were also taken into consideration. By use of the Kaplan-Meier and Stepwise regression analysis [29], as well as the t-test, we found that the co-expression of GEP100 and AMAP1 proteins both at high levels correlates with the rapidity of local recurrence after BCT.

## Materials and Methods

### Patient population

Four hundred eighty-three breasts of 479 breast cancer patients, who received breast conservation surgery followed by whole breast irradiation at the Hokkaido University Hospital between 1988 and 2008, were retrospectively analyzed. By May 2010, a total of 20 relapses in 20 patients were observed with a median follow-up of 54 months; and among them, specimens from 19 patients were available. In all but one patient, the breast was the first site of recurrence. The remaining one patient experienced initial recurrence at the regional lymph node at 4 months, and breast recurrence at 18 months after BCT. This study has been approved by the institutional review board of Hokkaido University Hospital (010-0203). The requirement for written consent was waived by our institutional board according to Ethical Guidelines for Clinical Studies of Japanese Ministry of Health, Labour and Welfare.

### Treatment methods

Eleven patients received lumpectomy, and 8 received quadrantectomy. Sixteen of 19 patients (84%) received axillary lymph node dissection and the other 3 (16%) received sentinel lymph node biopsy. One patient at the TNM stage (UICC6^th^ edition) of T3N1M0 received preoperative chemotherapy including Trastuzmab, and also received Trastuzmab after surgery. One patient received chemotherapy during surgery. One patient received chemotherapy both during and after surgery. Three patients received chemotherapy after radiotherapy. Five patients received hormone therapy after radiotherapy. All patients received tangential whole breast irradiation to the affected breast. One patient received irradiation in the parasternal and supraclavicular lymph node regions. Prescribed irradiation doses to patients with microscopically complete excision was 45 Gy in 18 fractions, and with microscopically incomplete excision was 50 Gy in 20 fractions. Since July 2005, patients younger than 50 years-old have received 50 Gy in 20 fractions irrespective of surgical margin status.

### Tissue specimens

All 19 pathological specimens were widely resected surgical specimens. Pathological features and surgical margin status were reviewed by a pathologist retrospectively in a blind manner (K.H). Margin status was defined as follows: positive margins as tumors (either invasive ductal carcinoma (IDC) or ductal carcinoma in situ (DCIS)) seen at the inked edges of the resection; close margins as tumors seen within 5 mm from, but not at the end of the resection edges; and negative margins as tumors not seen within 5 mm from the resection edges.

### Immunohistochemistry

Antibodies against AMAP1 [18] and GEP100 [19] were described previously. Antibodies against EGFR (31G7 mAb, Nichirei) and HER2 (A0485, Dako) were from commercial sources. Immunohistochemical staining was performed using 4 µm-thick formalin-fixed paraffin-embedded sequential sections, as follows. Samples were first deparaffinized in xylene and dehydrated in graded alcohols. After rinsing in TBS buffer (25 mM Tris-HCl (pH 7.4), 137 mM NaCl, 2.7 mM KCl), they were processed for antigen retrieval in sodium citrate buffer (pH 6.0) at 95 °C for 40 min (for HER2), in a pepsin solution (Nichirei) at 37 °C for 10 min (for EGFR), or in a 1 mM ethylenedyamine tetra acetic acid (EDTA) retrieval solution (pH 9.0) (45211 Nichirei) at 95 °C for 40 min (for AMAP1 and GEP100). Endogenous peroxidase was then blocked by incubating in 0.3% H_2_O_2_-methanol at room temperature (RT) for 10 min. After rinsing with TBS, sections were then incubated with primary antibodies against EGFR (1:50), HER2 (1:200), AMAP1 (1:500) or GEP100 (1:100) for 30 min, then with EnVisionTM (Dako) for 30 min, and finally with peroxidase-conjugated streptavidin (Vector Labs) for 50 min. After rinsing in TBS, the colouring reaction was performed with DAB (Dojin) for 5 min. Each section was counterstained with haematoxylin. These processes were performed at RT.

### Scoring

Immunohistochemical samples were scored by two pathologists (S.T. and M.T) independently in a blind fashion. Anti-EGFR staining was scored as 0 to 2+, in which staining of the non-cancerous ductal epithelia was considered as 1+. Anti-HER2 staining was scored as 0 to 3+, in which strong membrane staining of more than 30% of tumor cells was scored as 3+, weak to moderate membrane staining of 10-30% of tumor cells were as 2+, strong membrane staining in less than 10% of tumor cells were also as 2+, faint membrane staining of less than 10% of tumor cells was as 1+, and faint membrane staining with less than 10% of tumor cells was as 0. Anti-AMAP1 and anti-GEP100 staining were each scored as 1+ to 2+, in which staining of the non-cancerous ductal epithelia was scored as 1+.

### Statistical analysis

Stepwise regression analysis [29] was used in which the threshold of the p-value was set at 0.05. The log times to the local recurrence were compared between subgroups by the t-test. The Kaplan-Meier curves of each factor for the time to local recurrence were also calculated. These Analyses were performed using JMP^®^ Version 10 (SAS Institute).

## Results

### Characteristics of patients

Characteristics and methods of treatments of patients are summarized in Table 1. The pathological characteristics and time of local recurrence are shown in Table 2. Pathological tumor stages were Tis for 7 patients, T1 for 9 patients, and T2 for 3 patients. One of the 7 DCIS-patients who received chemotherapy before the operation, had a biopsy specimen which was diagnosed as IDC with a stage of T3. Fifteen patients were node-negative and 4 were node-positive. Twelve patients were surgical margin-negatives, while 3 were close margins and 4 were positive margins. Median time of local recurrence of these patients was 38 months (range 8-179 months).

**Table 1:** Patients characteristics and treatment methods.

Age	No. patient
30-39	2
40-49	9
50-59	5
60-69	2
70-	1
Surgery primary site	
Lumpectomy	11
Quadrantectomy	8
Surgery axillary node	
Sentinel lymph node biopsy	3
Axillary dissection	16
Radiation dose	
45 Gy/ 18 fractions	9
50 Gy/ 20 fractions	10
Chemotherapy	
Neoadjuvant chemotherapy	1
Intraoperative chemotherapy	1
Intraoperative and adjuvant chemotherapy	1
Adjuvant chemotherapy	3
Hormone therapy	5

**Table 2:** Clinical and pathological characteristics of the patients.

Case	p-stage	Histology	Surgical margin	ER	PgR	EGFR	HER2	AMAP1	GEP100	Months
1	yIIA	IDC	Negative	N	N	0	3	2	2	8
2	IIB	IDC	Negative	N	N	1	3	1	1	18
3	0	DCIS	Negative	P	P	0	1	2	2	19
4	0	DCIS	Intraductal close	N	N	1	3	2	2	20
5	0	DCIS	Intraductal positive	N	N	0	3	2	2	21
6	IA	IDC	Intraductal positive	P	P	0	2	1	1	21
7	0	DCIS	Negative	P	P	1	1	1	1	25
8	IA	IDC	Negative	P	P	0	2	1	1	29
9	IA	IDC	Negative	P	P	1	2	1	1	35
10	IIA	IDC	Intraductal close	P	P	0	2	1	1	38
11	IA	Invasive lobular ca.	Invasive lobular close	N	N	NA	2	1	1	42
12	0	DCIS	Negative	U	U	0	0	1	2	45
13	IA	IDC	Negative	U	U	0	1	1	2	61
14	0	DCIS	Negative	P	P	0	1	1	1	99
15	IIA	IDC	Intraductal positive	N	P	1	0	1	1	109
16	IIA	IDC	Negative	P	P	0	2	1	1	136
17	IA	IDC	Intraductal positive	P	P	0	3	1	2	124
18	IIA	IDC	Negative	N	N	0	2	1	2	160
19	IA	IDC	Negative	N	P	0	2	2	1	179

ER: estrogen receptor, PgR: progesterone receptor, EGFR: epidermal growth factor receptor, HER2: human epidermal growth factor receptor 2, Months: The time from completion of radiotherapy to recurrence, IDC: invasive ductal carcinoma, DCIS: ductal carcinoma in situ, P: positive, N: negative, U: unknown, NA: not applicable

### Clinical factors and time of local recurrence

**Figure 1:**
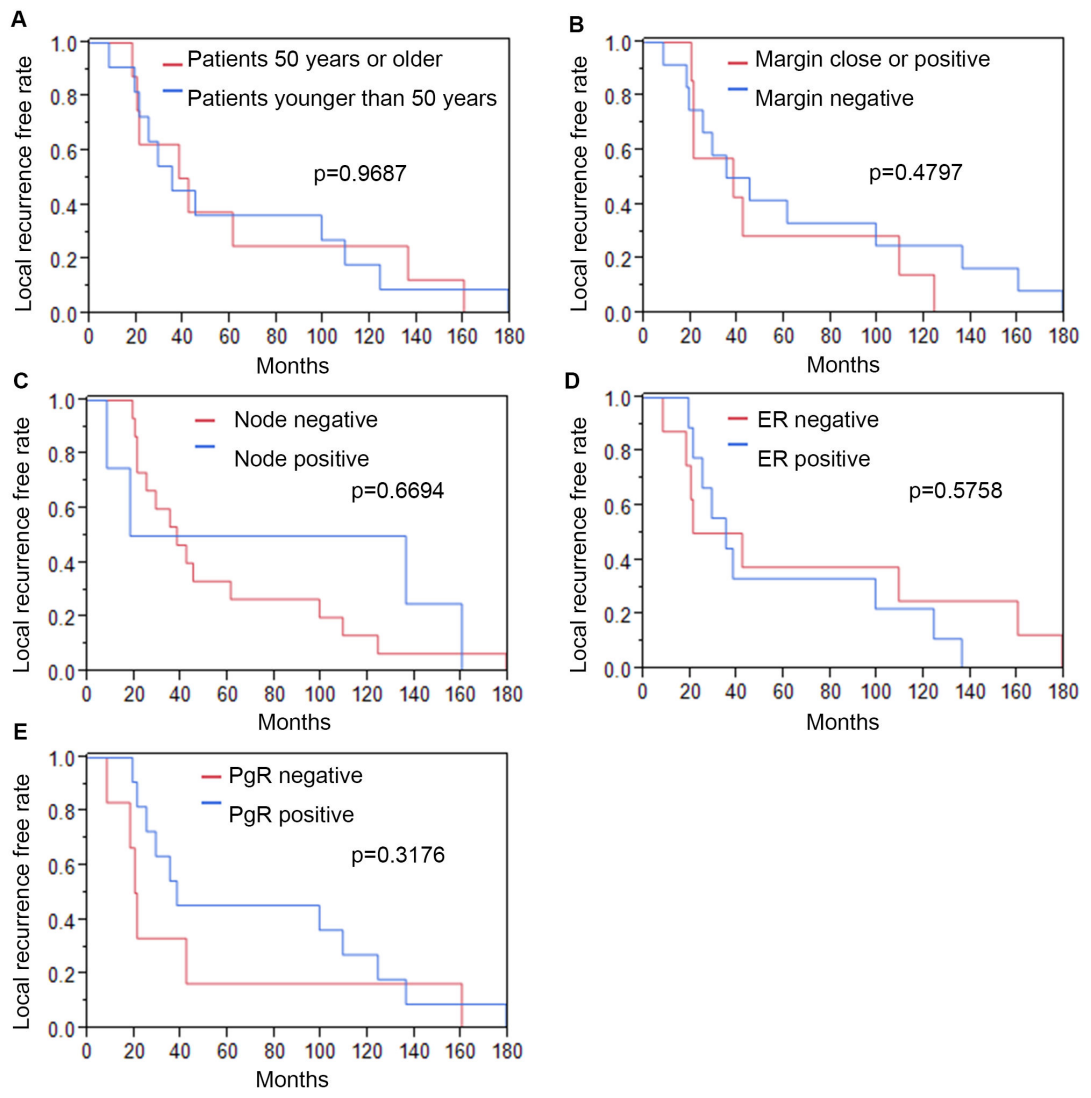
Clinical factors and time of local recurrence. A.-E. Kaplan-Meier curves for local recurrence-free survival after BCT, stratified by age (A), surgical margin status (B), node-positivity (C), ER status (D) and PgR status (E). Age, margin-status, nodal-status and hormone receptor- status did not correlate with the rapidity of local recurrence. Months: The time from completion of radiotherapy to recurrence. ER: estrogen receptor. PgR: progesterone receptor.

We first examined whether previously reported factors, such as age, surgical margin status, node-, ER-, and PgR-positivity, correlate with the time of local recurrence. We found that patients younger than 50 years-old showed a median value of 35 months for local recurrence-free survival, while those older than 50 years showed a median value of 40 months (Figure 1A). Patients with negative margins showed a median value of 40 months, while patients with close or positive margins showed 38 months (Figure 1B). Patients with node-positivity showed 77 months, while those with node-negativity showed 38 months (Figure 1C). Patients that were ER-positive showed 35 months, while those that were ER-negative showed 31.5 months (Figure 1D). Patients that were PgR-positive showed 38 months, while those that were PgR-negative showed 20.5 months (Figure 1E). Therefore, there was no statistical difference in the median time of local recurrence after BCT for age (p=0.97), margin status (p=0.48), node positivity (p=0.67), ER status (P=0.58) or PgR status (P=0.32), as calculated by the log-rank test.

### Co-expression of GEP100 and AMAP1 at high levels correlates with local recurrence

**Figure 2:**
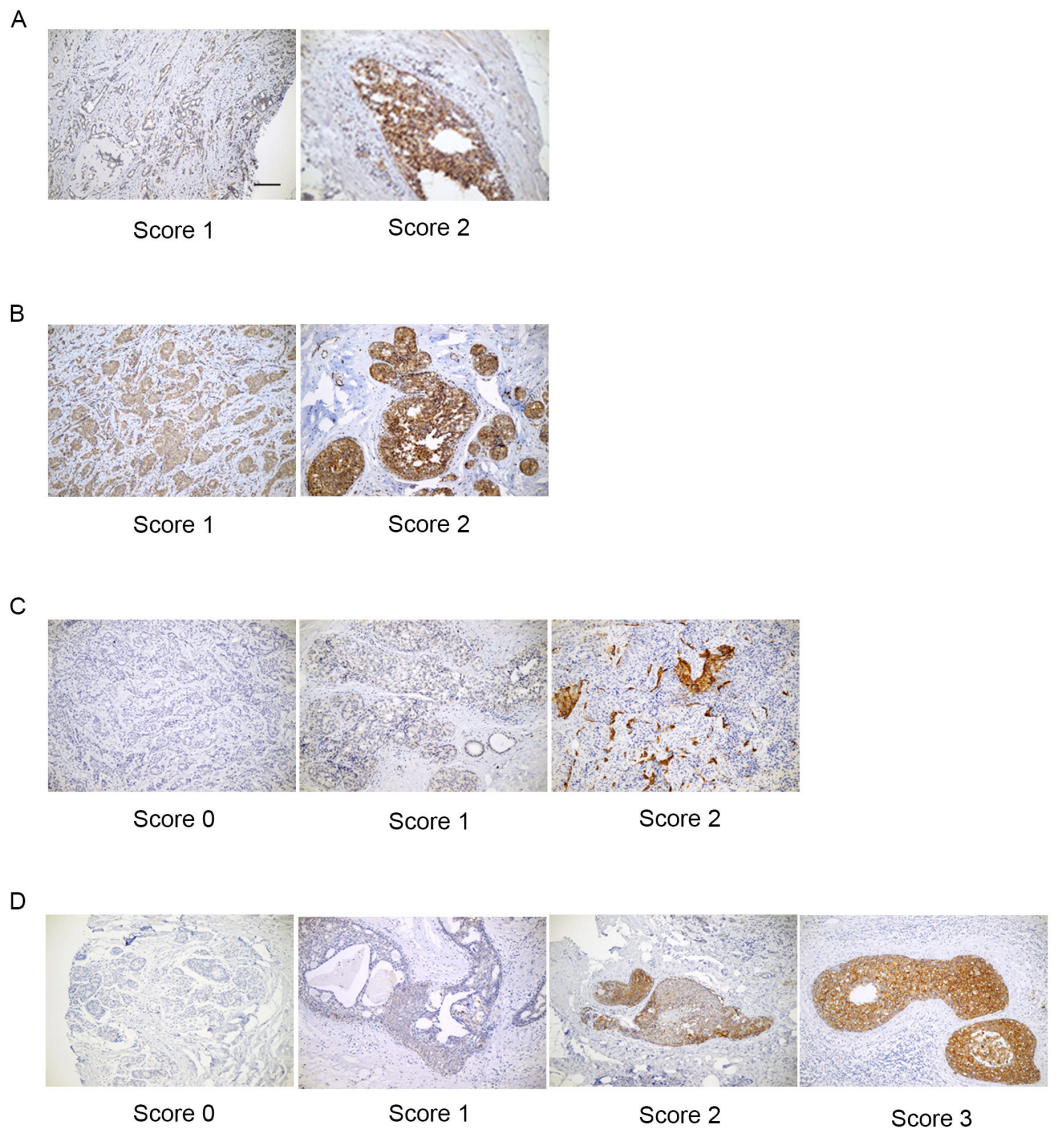
Immunohistochemical stainings of GEP100 (A), AMAP1 (B), EGFR (C) and HER2 (D). Representative figures are shown. Bars, 100 µm. EGFR: epidermal growth factor receptor. HER2: human epidermal growth factor receptor 2.

The GEP100-Arf6-AMAP1 pathway can be activated by RTKs, such as EGFR and Her2. We therefore next analyzed the expression of these proteins. For EGFR, 5 cases (27.7%) exhibited score 1+, while 13 cases (72.2%) were negative (score 0) and 1 case was not applicable (NA). For HER2, 5 cases (26.3%), 8 cases (42.1%), 4 cases (21.0%) and 2 cases (10.5%) were scored as 3+, 2+, 1+ and 0, respectively. For AMAP1, 5 cases (26.3%) were scored as 2+, while 14 cases (73.7%) were as 1+. For GEP100, 8 cases (42.1%) were scored as 2+, while 11 cases (57.9%) were as 1+. Representative images of the immunohistochemical staining are shown in Figure 2. On the other hand, like in the case of many other small GTP-binding proteins, antibodies against Arf6 applicable for immunohistochemistry were not available.

**Figure 3:**
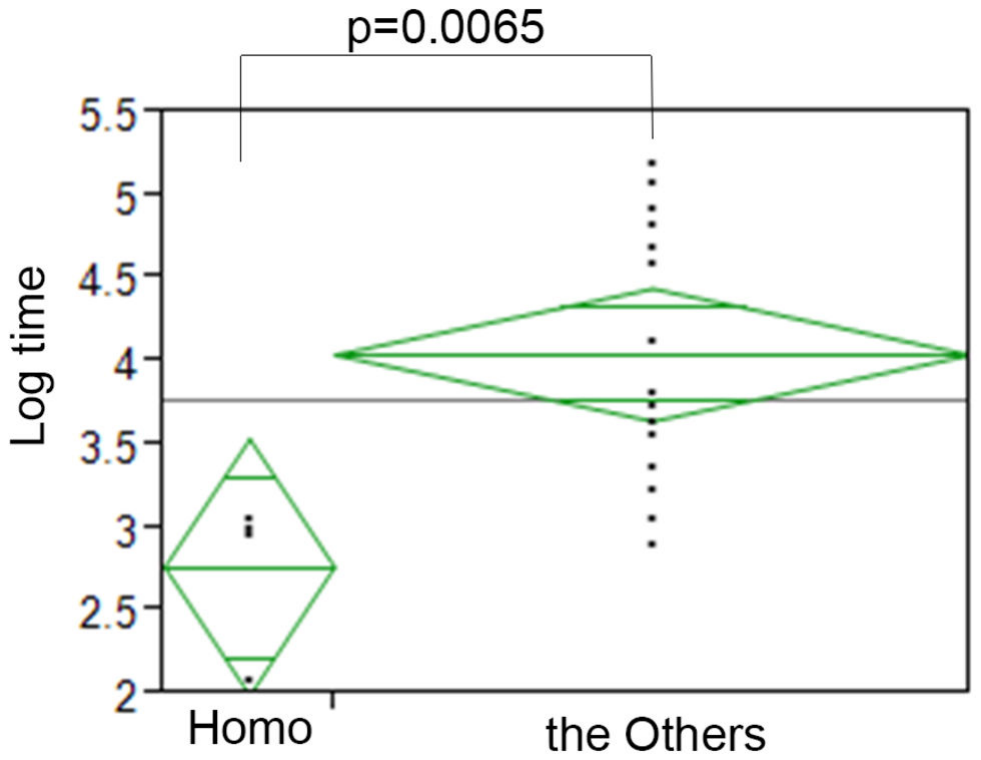
The t-test for the rapidity of local recurrence of subgroups of GEP100 and AMAP1. Mean and 95% confidence intervals of the natural logarism of months from completion of radiotherapy to the local recurrence for subgroups of GEP100 and AMAP1 expressions. Samples strongly positive for both GEP100 and AMAP1 (Homo group) show shorter times of local recurrence than the others (p=0.0065). The Homo group (GEP100 (2+) and AMAP1 (2+)) vs. the Others.

**Figure 4:**
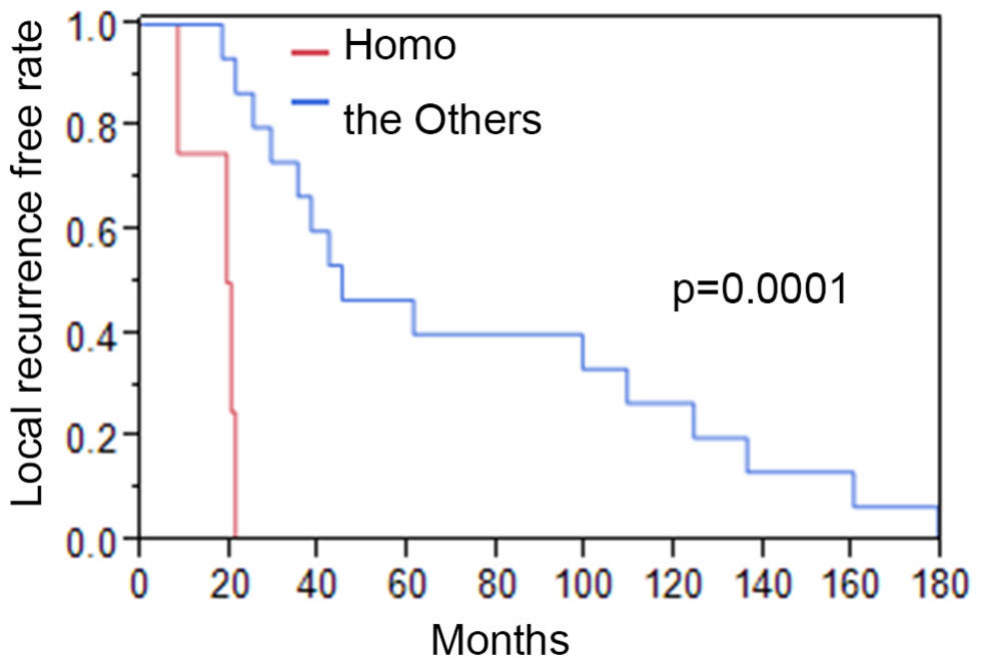
Kaplan-Meier curves for the time to event of subgroups of AMAP1 and GEP100. Homo group (GEP100 (2+) /AMAP1 (2+)) shows a shorter time to local relapse after the completion of BCT than the Others, with a p value of 0.0001. Months: The time from completion of radiotherapy to recurrence.

Stepwise regression analysis [29] then identified the expression of GEP100 and AMAP1, and their interaction, as factors associated with time to local recurrence with a p value of 0.0018. The t-test also showed that samples strongly positive for both GEP100 and AMAP1 (Homo group) show shorter times of local recurrence than the others, in which the p-value was calculated as 0.0065 (Figure 3). The Kaplan-Meier curves of the time from completion of BCT to local recurrence for the Homo group and the others also showed a statistical difference (p=0.0001, Figure 4). On the other hand, expression of GEP100 or AMAP1 on its own did not show such a significance (Figure S1 A, B). EGFR or HER2 on its own (Figure S1 C, D), or their co-expression with either GEP100 or AMAP1 also did not show any significance for the rapidity of local recurrence (Figure S1 E-H).

## Discussion

BCT for the early stages of IDC and DCIS provides excellent local control rates and survival of patients. However, there still exist significant populations of patients who experience local recurrence after BCT. In this study, we focused on a group of patients who developed local recurrence after BCT, and found that co-overexpression of GEP100 and AMAP1 proteins statistically correlates with the rapidity of local recurrence, even though in this cohort of patients surgical margin-status and ages were not correlated with the rapidity of local recurrence. On the other hand, expression of either GEP100 or AMAP1 alone did not correlate with the rapidity of local recurrence. These results are consistent with the notion that GEP100 and AMAP1 are components of the Arf6 pathway, and expression of either one of these proteins alone does not result in an active Arf6 pathway. Expression of EGFR, HER2, ER or PgR on its own also did not correlate with the rapidity of local recurrence in our samples. On the other hand, due to the relatively small number of patients who showed local recurrence in Hokkaido University Hospital during the past 20 years, we were unable to analyze the statistical significance as to whether the simultaneous expression of more than three of these proteins is correlated with local recurrence.

We are also interested in analyzing whether co-expression of these proteins correlates with distant metastasis. However, given that the 'Mamma-Print', a signature identified as being correlated with distant metastasis after BCT, does not precisely predict local recurrence [12], factors involved in the distant metastasis of breast tumors might be substantially different from those involved in local recurrence (also see below). Moreover, we have yet to investigate whether the co-expression of GEP100 and AMAP1 proteins correlates with the 'occurrence' of local recurrence.

Local recurrence occurs as a consequence of mixed and complicated genome alterations of tumor cells, as well as many different effects from their microenvironments. Nevertheless, it is conceivable to assume that 'dissemination of tumors cells before physical resection' and 'their radio-resistance' would be minimal pre-requisites for local recurrence after BCT, as mentioned earlier. The RTKs-GEP100-Afr6-AMAP1 pathway largely contributes to the moving out of tumor cells, and moreover regulates the recycling of β1 integrins [22,30]. We have observed that siRNA-mediated knockdown of GEP100, Arf6 and AMAP1 greatly enhances sensitivity to ionizing-radiation of MDA-MB-231 breast cancer cells (our unpublished results), in addition to the blockage of invasive and metastatic activities [17-19]. Therefore, presence of the Arf6 pathway would render tumor cells the potential to not only move-out from their original sites but also to be resistant to the ioninzing-radiation used in BCT.

Identification of gene expression signatures predictive for local recurrence after BCT have so far been unsuccessful, while, for example, gene expression signatures predictive for locoregional recurrence after mastectomy of breast cancer, which does not use radiotherapy coupled with physical resection, was reported [31]. Protein levels of Arf6 and AMAP1 do not correlate with their mRNA levels [17,18,32]; and indeed their mRNAs both have long 5'-UTRs with large free-energy changes and are classified to be typical 'weak-mRNAs' that are known to be inefficiently translated on their own. We have moreover found that p53 mutations and micro-RNAs are also involved in the expression of *Arf6* and *AMAP1* mRNAs (will be published elsewhere). Such properties and regulation of *AMAP1* and *Arf6* mRNAs might have hindered these mRNAs from being identified previously to be correlated with tumor malignancy and recurrence. Nevertheless, given that epigenetic events and cellular metabolic conditions are deeply involved in the expression of Arf6 and AMAP1, it is worthy to investigate whether factors and events exist within cells and the microenvironments that make transformed cells to express all the set of proteins of the RTKs-GEP100-Arf6-AMAP1 pathway simultaneously, and to be activated by external ligands. Such identification might contribute greatly to the further development of therapeutics to prevent and to treat the local recurrence of breast cancers.

## Supporting Information

Figure S1 A.-D. Kaplan-Meier curves of time to event of scoring GEP100 (GEP100 (1+) vs. GEP100 (2+)) (A), AMAP1 (AMAP1 (1+) vs. AMAP1 (2+)) (B), EGFR (EGFR (0) vs. EGFR (1+/2+)) (C) and HER2 (HER2 (0/1+) vs. HER2 (2+/3+)) (D), respectively.
There were no statistic difference in the rapidity of local recurrence for GEP100 (p=0.6634), AMAP1 (p=0.6847), EGFR (p=0.1584) and HER2 (p=0.7303).
E.-F. Kaplan-Meier curves for the time to event of subgroup of EGFR and GEP100 (EGFR (0)/GEP100 (1+) vs. the Others) (E), EGFR and AMAP1 (EGFR (0)/AMAP1 (1+) vs. the Others) (F). There were no statistic difference in the rapidity of local recurrence for subgroup of EGFR and GEP100 (p=0.2320), EGFR and AMAP1 (p=0.3009).
G.-H. Kaplan-Meier curves for the time to event of subgroup of HER2 and GEP100 (HER2 (2+/3+)/GEP100 (2+) vs. the Others) (G), HER2 and AMAP1 (HER2 (2+/3+)/AMAP1 (2+) vs. the Others) (H). There were no statistic difference in the rapidity of local recurrence for subgroup of HER2 and GEP100 (p=0.9554), HER2 and AMAP1 (p=0.9040).
Months: The time from completion of radiotherapy to recurrence.
EGFR: epidermal growth factor receptor.
HER2: human epidermal growth factor receptor 2.
(TIF)
