# Supplementary figures and images for: Co-Overexpression of GEP100 and AMAP1 Proteins Correlates with Rapid Local Recurrence after Breast Conservative Therapy
Source: PLoS One. 2013 Oct 7;8(10):e76791. doi: 10.1371/journal.pone.0076791 (PMC3792161; doi:10.1371/journal.pone.0076791)

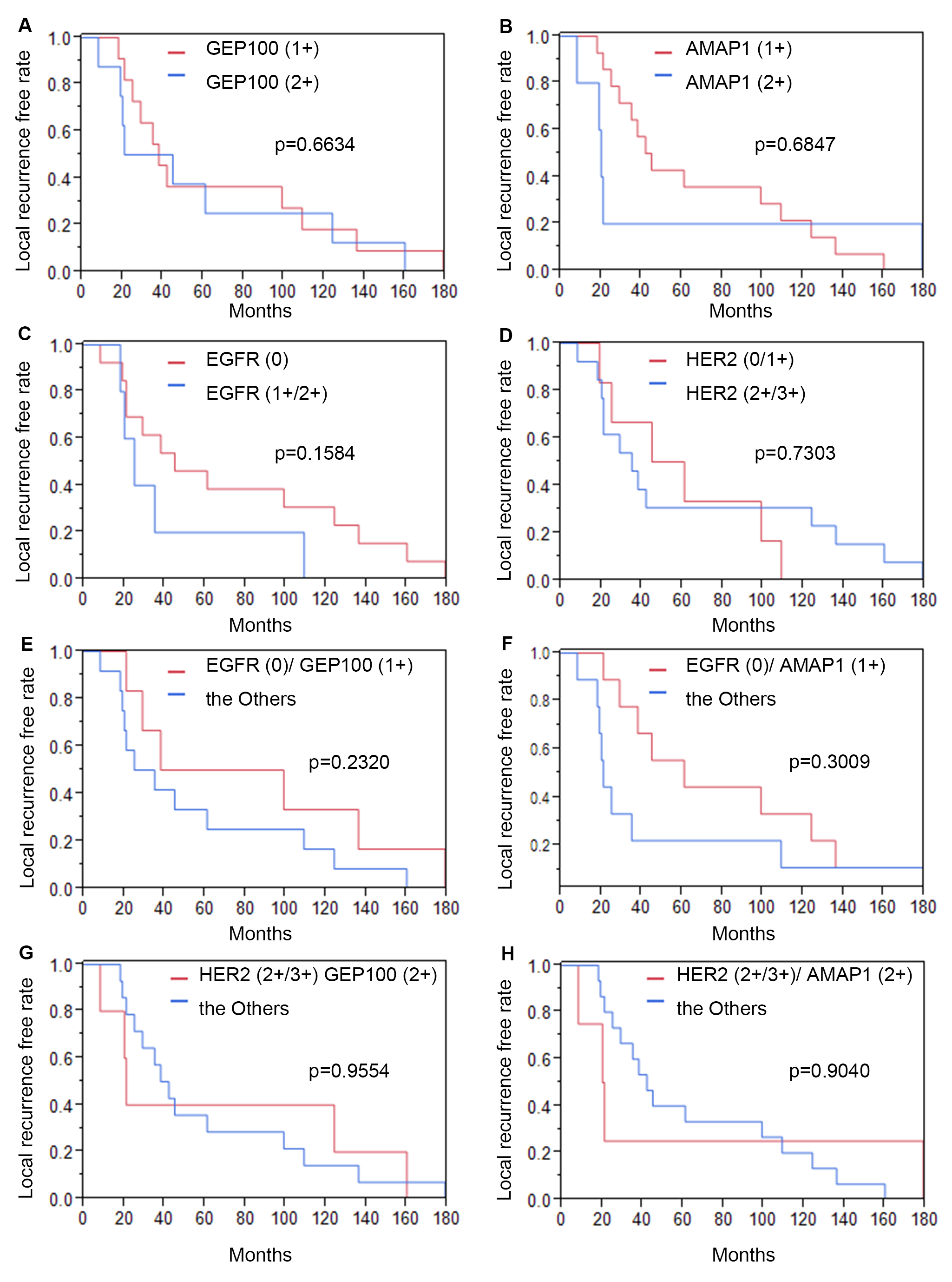

Supplement: Figure S1 — A.-D. Kaplan-Meier curves of time to event of scoring GEP100 (GEP100 (1+) vs. GEP100 (2+)) (A), AMAP1 (AMAP1 (1+) vs. AMAP1 (2+)) (B), EGFR (EGFR (0) vs. EGFR (1+/2+)) (C) and HER2 (HER2 (0/1+) vs. HER2 (2+/3+)) (D), respectively. There were no statistic difference in the rapidity of local recurrence for GEP100 (p=0.6634), AMAP1 (p=0.6847), EGFR (p=0.1584) and HER2 (p=0.7303). E.-F. Kaplan-Meier curves for the time to event of subgroup of EGFR and GEP100 (EGFR (0)/GEP100 (1+) vs. the Others) (E), EGFR and AMAP1 (EGFR (0)/AMAP1 (1+) vs. the Others) (F). There were no statistic difference in the rapidity of local recurrence for subgroup of EGFR and GEP100 (p=0.2320), EGFR and AMAP1 (p=0.3009). G.-H. Kaplan-Meier curves for the time to event of subgroup of HER2 and GEP100 (HER2 (2+/3+)/GEP100 (2+) vs. the Others) (G), HER2 and AMAP1 (HER2 (2+/3+)/AMAP1 (2+) vs. the Others) (H). There were no statistic difference in the rapidity of local recurrence for subgroup of HER2 and GEP100 (p=0.9554), HER2 and AMAP1 (p=0.9040). Months: The time from completion of radiotherapy to recurrence. EGFR: epidermal growth factor receptor. HER2: human epidermal growth factor receptor 2. (TIF) [file pone.0076791.s001.tif]
